# Mosquitoes Know No Borders: Surveillance of Potential Introduction of *Aedes* Species in Southern Québec, Canada

**DOI:** 10.3390/pathogens10080998

**Published:** 2021-08-07

**Authors:** Anne-Marie Lowe, Karl Forest-Bérard, Richard Trudel, Ernest Lo, Philippe Gamache, Matthieu Tandonnet, Serge-Olivier Kotchi, Patrick Leighton, Antonia Dibernardo, Robbin Lindsay, Antoinette Ludwig

**Affiliations:** 1 Direction des Risques Biologiques et de la Santé au Travail, Institut National de Santé Publique du Québec, 190 Boulevard Crémazie Est, Montréal, QC H2P 1E2, Canada; anne-marie.lowe@phac-aspc.gc.ca (A.-M.L.); richard.trudel.biol@outlook.com (R.T.); 2 Groupe de Recherche en Épidémiologie des Zoonoses et Santé Publique (GREZOSP), Faculty of Veterinary Medicine, University of Montréal, 3200 Rue Sicotte, Saint-Hyacinthe, QC J2S 2M2, Canada; serge-olivier.kotchi@phac-aspc.gc.ca (S.-O.K.); patrick.a.leighton@umontreal.ca (P.L.); antoinette.ludwig@canada.ca (A.L.); 3 Bureau D’information et D’études en Santé des Populations, Institut National de Santé Publique du Québec, 190 Boulevard Crémazie Est, Montréal, QC H2P 1E2, Canada; ernest.lo@inspq.qc.ca (E.L.); Philippe.Gamache@inspq.qc.ca (P.G.); matthieu.tandonnet@inspq.qc.ca (M.T.); 4 Department of Epidemiology, Biostatistics and Occupational Health, McGill University, 1020 Pine Ave. West, Montréal, QC H3A 1A2, Canada; 5 Public Health Risk Sciences Division, National Microbiology Laboratory, Public Health Agency of Canada, 3200 Rue Sicotte, Saint-Hyacinthe, QC J2S 2M2, Canada; 6 Department of Pathology and Microbiology, Faculty of Veterinary Medicine, University of Montréal, 3200 Rue Sicotte, Saint-Hyacinthe, QC J2S 2M2, Canada; 7 Zoonotic Diseases and Special Pathogens Division, National Microbiology Laboratory, Public Health Agency of Canada, 1015 Arlington St., Winnipeg, MB R3E 3M4, Canada; antonia.dibernardo@canada.ca (A.D.); robbin.lindsay@canada.ca (R.L.)

**Keywords:** invasive mosquito species, public health, *Aedes albopictus*, *Aedes aegypti*, *Ochlerotatus japonicus*, *Ochlerotatus triseriatus*

## Abstract

Current climatic conditions limit the distribution of *Aedes* (*Stegomyia*) *albopictus* (Skuse, Diptera: Culicidae) in the north, but predictive climate models suggest this species could establish itself in southern Canada by 2040. A vector of chikungunya, dengue, yellow fever, Zika and West Nile viruses, the *Ae. Albopictus* has been detected in Windsor, Ontario since 2016. Given the potential public health implications, and knowing that *Aedes spp.* can easily be introduced by ground transportation, this study aimed to determine if specimens could be detected, using an adequate methodology, in southern Québec. Mosquitoes were sampled in 2016 and 2017 along the main roads connecting Canada and the U.S., using Biogent traps (Sentinel-2, Gravide *Aedes* traps) and ovitraps. Overall, 24 mosquito spp. were captured, excluding *Ae. Albopictus*, but detecting one *Aedes (Stegomyia) aegypti* (Skuse) specimen (laid eggs). The most frequent species among captured adults were *Ochlerotatus triseriatus*, *Culex pipiens* complex, and *Ochlerotatus japonicus* (31.0%, 26.0%, and 17.3%, respectively). The present study adds to the increasing number of studies reporting on the range expansions of these mosquito species, and suggests that ongoing monitoring, using multiple capture techniques targeting a wide range of species, may provide useful information to public health with respect to the growing risk of emerging mosquito-borne diseases in southern Canada.

## 1. Introduction

Climate change modifies mosquito species distributions around the world, enabling the establishment of newly introduced species. Invasive mosquito species (IMS) are newly introduced species in an area, where they tend to spread, potentially having an impact on native species and ecosystems or on human activities (agriculture, conservation, and tourism) [1,2]. Mosquito species invasion is a global concern. In Europe, IMS represent a public health threat in an increasing number of countries [3,4], while in the Americas, the West Nile virus (WNV) epidemic, which started in 1999 in the state of New York [5], appeared to be transmitted by an invasive species, *Culex pipiens* (Linnaeus, 1758). 

The 2016 Zika epidemics renewed concerns in North America about the presence of locally established vector populations, namely of the yellow fever mosquito *Aedes* (*Stegomyia*) *aegypti* (Linnaeus, 1762) and the Asian tiger mosquito *Aedes* (*Stegomyia*) *albopictus* (Skuse, 1894) (Diptera: Culicidae). Among the IMS currently present in North America, these two species represent specific threats for Canada. Prior to 2016, *Ae. albopictus* had been detected only on a rare and episodic basis in Canada by the WNV surveillance program, near the Montréal international airport in Québec [6,7] and in southern communities of Ontario [8]. In 2017, the Centers for Disease Control and Prevention (CDC) of the United States of America estimated that *Ae. albopictus*’ potential distribution in the United States could extend to the northernmost states of the east coast, which share borders with Canada: New York, Vermont, and New Hampshire [9,10,11]. Enhanced surveillance in Windsor-Essex County (in Ontario, Canada) detected the presence of both species at the larval stage in 2016. This was the first time *Ae. aegypti* was documented in Canada, and the first official detection of juvenile stages for either species north of the border [8]. Diapause is a key adaptation for winter survival of mosquitoes [12]. While both species are highly invasive and competitive anthropophilic container-inhabiting species, neither *Ae. albopictus* nor *Ae. aegypti* are particularly good at overwintering. While the former is capable of ecological plasticity to some extent, it has limited egg diapause and cold-hardiness in North America [13,14], which has restricted its northward range expansion during the last 35 years to latitudes well south of Québec [11].

The spread of these invasive species is a major public health concern, mainly because of their ability to transmit a variety of arboviruses. Indeed, both species are competent vectors of the chikungunya virus, all four dengue virus serotypes, yellow fever virus, and Zika virus [10,13,15,16,17]. Additionally, *Ae. albopictus* has been shown, under experimental conditions, to be a competent vector for at least 22 arboviruses alone, including some that are endemic to Canada, such as WNV, Cache Valley, eastern equine encephalitis, and Jamestown Canyon viruses [18,19,20], thus favoring a potential resurgence of mosquito-borne diseases native to North America [21,22,23]. In addition, *Ae. albopictus* is known to thrive around human habitation and urbanized environments, and to be a significant biting nuisance, feeding on a wide range of hosts [24].

Other medically-relevant mosquito species ecologically-related to invasive *Aedes spp.* are already present in Canadian territory, namely *Ochlerotatus japonicus* [25], sometimes referred to as *Aedes japonicus,* a highly invasive species in North America, which was first detected in Quebec in 2002, and a competent vector of the WNV and Cache Valley virus [26,27,28,29]. *Ochlerotatus triseriatus* is endemic to the eastern part of North America, and was detected in Manitoba, Canada [30]. It is a primary vector of La Crosse encephalitis virus (LACV), and is competent for other arboviruses, including WNV [31,32,33,34]. Along with *Ae. albopictus*, *Oc. hendersoni*, and *Oc. atropalpus*, they form the *Aedine Multivoltine group, Oc. triseriatus Type* (AMOT) ecological group, characterized by their use of naturally-occurring or artificial water containers for egg laying [34]. Eggs of *Oc. triseriatus* and *Oc. japonicus* can undergo diapause and, thus, overwinter in temperate climates, despite adults being unable to survive through this period [35,36]. While there are temporal differences among these species’ life cycles, they are far from distinct and they frequently co-occur, cohabitating in an “*Aedes*/*Ochlerotatus* community” [13,26,31,34,37,38,39].

To the best of our knowledge, no specific surveillance or study has been conducted on the AMOT species and their role in Québec’s arbovirus circulation, although specimens were detected at low abundance through WNV surveillance [6]. Québec’s mosquito surveillance was historically designed for monitoring WNV and targets its most abundant competent vector species, namely the *Culex pipiens* complex and *Ae. Vexans* (Meigen, 1830). 

For targeted surveillance of *Ae. albopictus* and *Ae. aegypti*, we aimed to develop a sampling design that focused on the main introduction pathways for these exotic mosquitoes. Vehicular movements along road networks have been identified as the main route by which adult mosquitoes move to new regions in Europe [40,41,42,43]. The European Centre for Disease Prevention and Control recommends different surveillance scenarios to improve monitoring approaches and to limit the accidental mechanical introduction of IMS. Scenario 1 (unreported and unestablished IMS, with non-negligible introduction and establishment potential) corresponds to the situation in Québec relative to invasive *Aedes spp.* and involves implementing surveillance aimed at detecting possible introduction and establishment of IMS at points of entry (sites where these mosquitoes could be introduced either by plane, boat, train or vehicle) [42]. Similarly, the World Health Organization’s guiding principles for entomological surveillance of *Aedes spp.* in the context of Zika virus epidemics recommend the enhancement of mosquito surveillance at border areas, and the implementation of vector surveillance and control at points of entry as per International Health Regulations [44,45]. 

Given the current situation in neighboring Ontario, and knowing that the mechanical introduction of IMS through ground transportation is well documented even over long distances, the objective of this work was to assess, using an appropriate surveillance design, whether invasive *Aedes spp.* (*Ae. albopictus* and *Ae. aegypti*), could be or have been introduced mechanically in southern Québec. We also aimed to characterize the presence and abundance of other species already established in this region, including *Oc. triseriatus* and *Oc. japonicus.*

## 2. Results

### 2.1. Mosquito Captures

In 2016, eggs were found in 10 ovitraps (OVI, designed to collect mosquito eggs, consist of a wood piece, as an oviposition substrate, placed in a black plastic bucket half-filled with a fermented herb solution, as a natural attractant) (OVI positivity index, OPI = 4%) and there was a positive OVI found at 7 of the 23 sampling sites (30.4%). A total of 472 eggs were retrieved from the OVI, of which 205 hatched, reached fourth instar larvae stage (43.4%), and were successfully identified to the species level. Four adult mosquitoes were accidentally captured in OVI and subsequently identified to the species level (Table 1). In 2017, eggs were found in 74 OVI (OPI = 22.8%), distributed over 11 of 12 sites (91.7%). A total of 7654 eggs were collected, of which 2206 hatched into larvae (28.8%); 1063 reached the adult stage (13.9% of the total eggs), and were identified by the species. The number of eggs collected daily peaked during mid-July to mid-August 2017, reaching a maximum of 3150 eggs on 31 July. Location-wise, the overall OPI was 62.5% (15 of 24 sites collected eggs during the two-year sampling period).

In 2016, out of the 46 Biogent Sentinel 2 (BGS2) trap-nights, 21 were adult-positive (adult trap positivity index, ATPI = 45.7%) and 30 of 276 of the Gravid *Aedes* traps (GAT) trap-nights were adult positive (ATPI = 10.9%). In 2017, out of the 108 BGS2 trap-nights, 55 were adult-positive (ATPI = 50.9%), and 42 of 324 of the GAT trap-nights were adult positive (ATPI = 13.0%). The number of adults captured peaked during the mid-July to mid-August 2017 period. All adults were identified to the species level in 2016 and five were not identified in 2017 because of sample deterioration. No mosquito (egg or adult) were found at sites one and two, located near Montréal, over the sampling period (2016–2017).

### 2.2. Molecular Identification of Eggs 

A total of 184 unhatched mosquito eggs (n = 85 in 2016 and n = 278 in 2017) were identified at the national microbiology laboratory (NML) based on analysis of the CO1 gene: *Oc. japonicus* (n = 41 in 2017), *Oc. triseriatus* (n = 84 in 2016 and n = 57 in 2017), *Oc. punctor* (n = 1 in 2016), and one invasive *Aedes spp*. (n = 1 in 2017, with 100% identity with *Ae. aegypti*, 99% identity with *Ae. albopictus*) (Table 1). The PCR and sequencing results of the CO1 gene on the *Aedes* mosquito were reproducible, with the PCR and sequencing performed twice, obtaining the same results. In addition, sequence data arising from the amplification of the ITS1 and ITS2 regions, and the results of a real-time PCR assay were most consistent with *Ae. aegypti*.

The *Ae. aegypti* egg came from an OVI deployed on 17 July 2017, at site no. 13, which was located in Saint-Armand (in Estrie, GPS coordinates: 45.0167, -73.0836) near the border between Québec and the state of Vermont. The OVI was on plot 3, located at the edge of a forested area, about 30 m away from the commercial road coming into Canada from the U.S., where trucks stop to pass border control. Overall, 85 eggs were captured during this OVI-night; five hatched and, among those, one could be identified by PCR as *Oc. triseriatus* (data not shown). Of the remaining 80 unhatched eggs sent to the national microbiology laboratory, 43 were too damaged to produce an amplification product, 36 were identified as *Oc. triseriatus*, and one was *Ae. aegypti*.

### 2.3. Mosquito Species Richness

Mosquito species richness indices (MSRI) were calculated yearly and monthly using egg and adult presence data: in total, 12 species were collected in 2016, 18 in 2017, and 24 for both 2016–2017 combined. Total MSRI were also calculated for each type of trap. BGS2 had a richness of eight in 2016, 17 in 2017, and 20 for both years combined. GAT had a richness of seven in 2016, five in 2017, and nine for both years combined. OVI had a richness of three in 2016, three in 2017, and four for both years combined (excluding species identified from occasional adult captures). The highest monthly MSRI were obtained in July 2017 (MSRI = 12) for BGS2, in August 2016 (MSRI = 6) for GAT, and in July 2017 (MSRI = 4) for OVI. 

### 2.4. Distribution of AMOT Species

#### 2.4.1. Spatial Distribution

**Figure 1:**
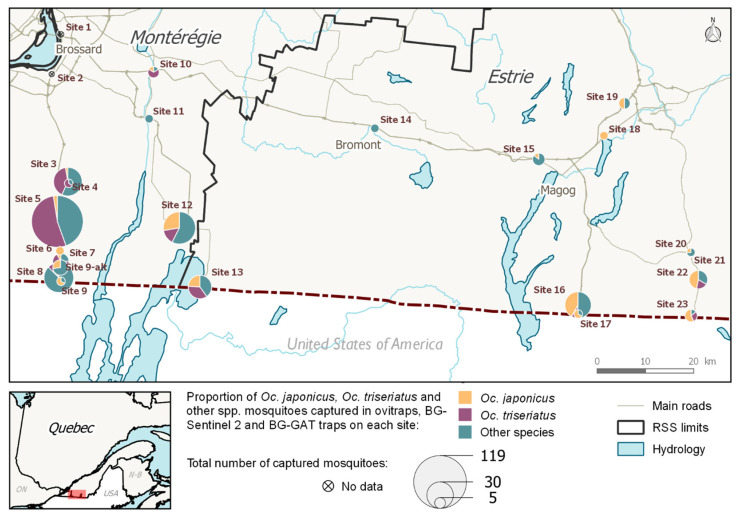
Map of the proportion of *Oc. japonicus*, *Oc. triseriatus*, and other species captured by adult traps at every site (2016 and 2017). GPS coordinates are listed in Table 2.

**Figure 2:**
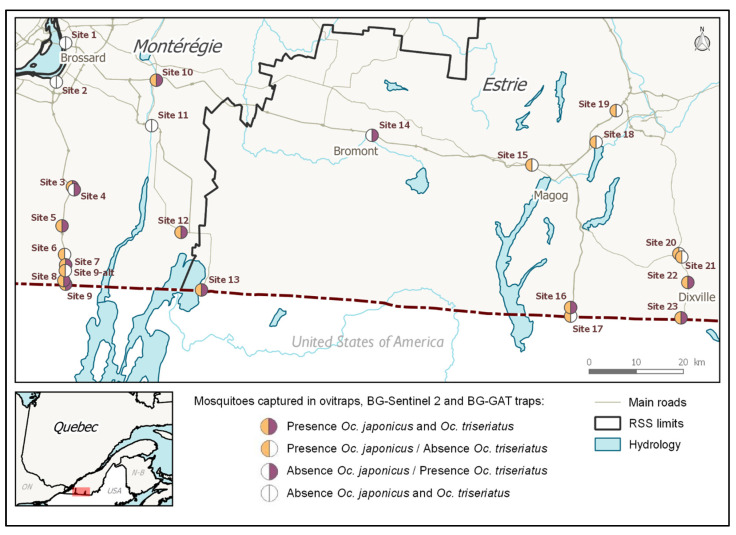
Map of the presence of *Oc. japonicus* and *Oc. triseriatus* in sampled sites, 2016 and 2017 (OVI, BGS2, and GAT samples). GPS coordinates are listed in Table 2.

Of the 11 sites located along the eastern roads running through Estrie (sites nos. 13–23), three were *Oc. triseriatus* dominant, and eight were *Oc. japonicus* dominant. *Oc. japonicus* was in a higher proportion at these four sites (Figure 1). The proportion of adult mosquitoes collected by species varied from site to site (aggregated by trap and by site). Of the 13 sites located along the two western roads running through Montérégie (sites nos. 1–12, including 9 and 9-alt), both *Oc. triseriatus* and *Oc. japonicus* were present (D > 5%) in eight sites. *Oc. triseriatus* occurred in higher proportion in four of the sites, while *Oc. japonicus* occurred in higher proportion in two of the sites (Figure 2). 

AMOT species presence distribution at 24 sites were estimated using combined egg and adult samples (Figure 2). *Oc. japonicus*’ was widespread: it was present in 19/24 or 79.2% of the sites sampled in 2016 and 2017, while *Oc. triseriatus* had a moderate presence (13/24, 54.2%). *Oc. japonicus* was present in 10/13 sites (76.9%) located along the two western roads running through Montérégie, while *Oc. triseriatus* was present in nine of these sites (69.2%). *Oc. japonicus* was present in 9/11 sites (81.8%) located along the eastern roads running through Estrie, and *Oc. triseriatus* was present in four (36.4%) of the eastern sites. The co-occurrence of both species was observed in 8/13 sites (61.5%) located along the roads running through Montérégie and on 3 of the 11 sites (27.3%) located along the roads running through Estrie. This co-occurrence of *Oc. japonicus* and *Oc. triseriatus* was more common in the western part of our study region than in the eastern part. 

#### 2.4.2. Temporal Distribution

**Figure 3:**
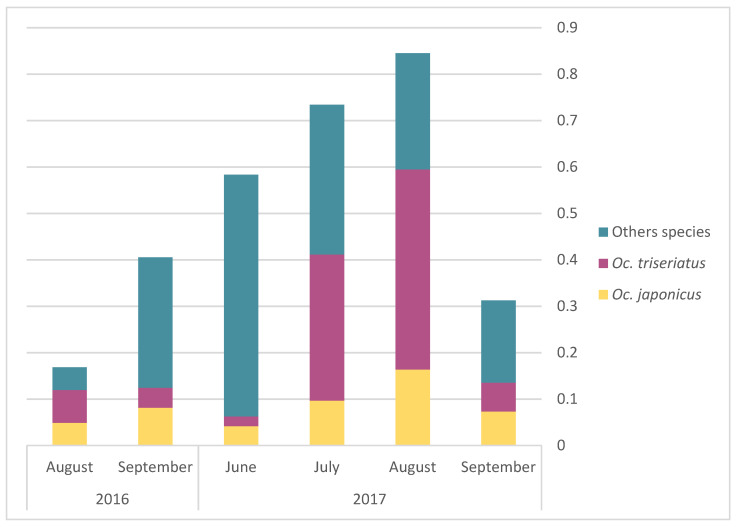
Monthly average of abundance of adult *Oc. japonicus*, *Oc. triseriatus*, and other species, by year.

**Figure 4:**
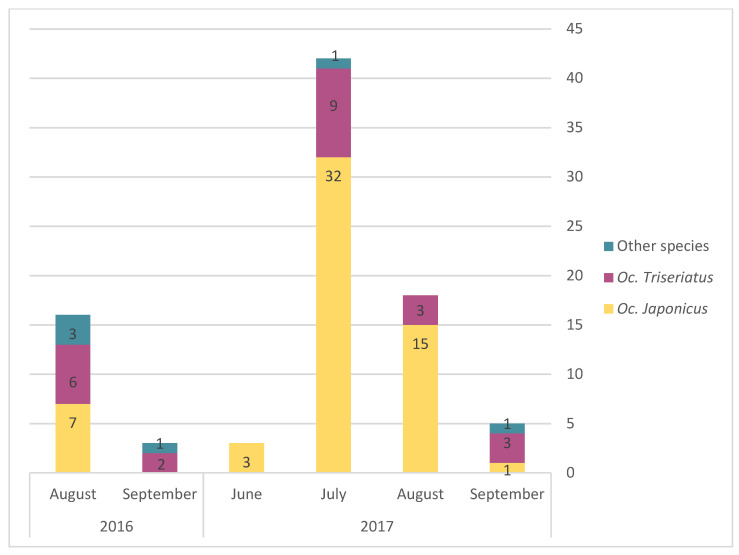
Relative frequency of presence of eggs of *Oc. japonicus, Oc. triseriatus*, and other species, by month and year.

The monthly average abundance of adult mosquito peaked in August for both *Oc. triseriatus* and *Oc. japonicus* (Figure 3). Egg presence for the different AMOT species varied following similar temporal trends, but with a peak in July (Figure 4). However, we observed a slight difference in terms of the relative frequency for *Oc. triseriatus* and *Oc. japonicus* between August and September: the majority of eggs captured in September were *Oc. triseriatus* in 2016 and 2017, while the majority of eggs captured in June to August were *Oc. japonicus* in 2016 and 2017 (Figure 4). The relative frequency of eggs peaked one month earlier than adults for both species (Figure 4).

The temporal consistency of the number of eggs of *Oc. japonicus* at the same site between 2016 and 2017 was assessed (Fisher’s exact test (FET), *p* = 0.005). The null hypothesis, according to which the abundance of eggs in certain sites is the same from one year to the other, was rejected. In other words, the abundance of *Oc. japonicus* eggs at the same sites appears to vary from one year to another. The same test, done for *Oc. triseriatus*, gave a *p*-value of 0.0667, suggesting that the presence of *Oc. triseriatus*’ eggs in certain sites is relatively constant from one year to another. The same comparison was done for adults’ presence and the results gave a *p*-value of 0.49 for *Oc. japonicus* and a *p*-value of 0.05 for *Oc. triseriatus*. This, again, showed that the adults of *Oc. triseriatus* seemed to be present in a relatively stable manner on the same sites one year after another, whereas adults of *Oc. japonicus* were not. 

### 2.5. Impact of Land Use on Mosquito Species Richness and Abundance

Regression analysis of species richness within sampling sites versus the percentage of developed zones and road segments (RR = 0.658, *p*-value < 0.001 and 0.440, *p* = 0.001, respectively) showed that species richness increased with decreasing development or shorter road segments. Species richness of the 24 sites was also positively associated with the degree of wetlands cover (RR = 1.12, *p* < 0.001) (Table 3).

The number of *Oc. triseriatus* adults captured using GAT was negatively associated with the percentage of developed zones (RR = 0.457, *p* = 0.002) (Table 4a); with a greater number of *Oc. triseriatus* being found in less developed zones. The number of *Oc. japonicus* adults was negatively associated with the road segments variable (RR = 0.386, *p* = 0.0158) (Table 4b), with a greater number of adults of this species being found where there were fewer road segments (or shorter total road segment length). The presence of *Oc. triseriatus* adults, captured using GAT and OVI eggs data, was statistically associated with developed areas (OR = 0.932, *p* < 0.001) and road segments (OR = 0.932, *p* = 0.03) (Table 5a). The presence of *Oc. japonicus* was statistically associated with the percentage of wetlands (OR = 1.013, *p* = 0.02), developed areas (OR = 0.973, *p* = 0.007), and road segments (OR = 0.923, *p* = 0.004) (Table 5b). While statistically significant, the magnitude of the effects detected is weak, with the OR values being close to one. 

## 3. Discussion

This study describes the first targeted monitoring of invasive *Aedes* mosquitoes in the province of Québec. We detected an egg of *Ae. aegypti* and a relative high abundance of *Oc. triseriatus* in southern Québec. These results are important for public health because (1) they demonstrate that vectors for well-known human diseases circulate actively in southern Québec, (2) that new invasive vectors have the potential to be introduced, and (3) that targeted surveillance of mosquito fauna can permit invasive species detection.

### 3.1. Detection of Ae. aegypti

No *Ae. albopictus* (at any stage) were found during the course of this project. However, *Ae. aegypti* was detected at egg stage. A single identified egg is not sufficient evidence to demonstrate establishment, but is rather the proof of a fortuitous, unlikely-yet-possible event, where an individual gravid female mosquito most-probably hitchhiked its way to Québec via ground-transportation, and laid her eggs once released into nature in one of the traps set there. This finding is in line with the ECDC’s assumption that a higher risk exists at the first stop or rest facility in a country, as many tourists stop there to buy local products at local prices (ECDC, 2012). While the overall capture ratio of IMS for this particular study is low, on the date of the capture, the mean temperature monitored on site for the sampling period was 21.2 °C (with a minimum of 16.6 °C and a maximum of 28.7 °C) (temperature probe, data not shown). This temperature is greater than the biting-threshold temperature for this species, which is 14 °C as reported by Brady et al [46]. Given the fact that *Ae. albopictus* has a lower temperature threshold for adult survival (13 °C for behavioral impairment and 9 °C for the end of adult activity) [46,47], this species could follow the same path as *Ae. aegypti* and potentially survive, at least during summer months. This is what has been observed in southern Ontario [8] and in the northern U.S. [48]. Recent modelling studies have explored the conditions that could determine the northern limit of the *Ae. albopictus* and *Ae. aegypti* extended range [49]. Results showed that this northern range distribution would be defined by two predictors: winter (December–February) cumulative degree-days > 10 °C and precipitation during the driest months [49]. The modeled distribution of *Ae. albopictus* was predicted to reach Québec’s southern border, whereas the distribution of *Ae. aegypti* was limited to American counties up to New Jersey. However, Johnson et al. (2017) stressed the fact that both species may be introduced, via accidental transport of eggs or during the immature stages, and that enhanced surveillance efforts are needed [49]. Even though the estimated limit of *Ae. aegypti* distribution is far from Québec’s southern border, the distance is compatible with transboundary ground transportation activities. These two studies (the only ones available for North America at the moment) also show that part of Canada is currently suitable for the *Ae. albopictus* mosquitoes and will be even more suitable in the future. Under actual winter climate conditions however, it is unlikely that the diapausing eggs of *Ae. albopictus* could survive in southern Québec, even under an adequate snow cover [50]. This finding represents only a fraction of all the sampled eggs in the course of this project, and the detection rate could potentially be increased with a sustained, intensive sampling. More monitoring needs to be done to further document this and keep track of the potential introduction of *Aedes* invasive species in the province. 

### 3.2. Detection and Abundance of Oc. Triseriatus and Oc. Japonicus

In Canada, *Oc. triseriatus* is known to be the primary vector of LACV (also transmitted by *Ae. albopictus*), the primary cause of viral encephalitis in children in the USA, with cases distributed mainly in the eastern and midwestern states [31,51]. Québec’s medical entomologists have been concerned about LACV for at least 35 years [52], but no LACV-associated clinical cases have yet been reported in Canada [53]. Prior to this study, it was unclear how abundant this species actually was in Québec and how widely it was dispersed. Indeed, since 2000, only small numbers of *Oc. triseriatus* were detected at regular frequency during the WNV mosquito surveillance operations in Québec using CDC light traps [6]. In 2016, these samples represented only 1% of all mosquitoes collected. However, *Oc. triseriatus* was extensively captured in this study, which relied on different trapping methodologies (accounting for 31% of all adult captures in GAT and BGS2 traps). These findings, along with others [54], suggest a near-ubiquitous presence of the species in southern Quebec, way superior to what was originally thought. Knowledge that LACV circulates in the USA, close to its border with Canada, and that the virus’ main vector is found across parts of southern Québec, is a first step to guiding the risk assessment of vector-borne transmission of this virus in a climate change context.

Following the same train of thought, *Oc. japonicus* [33] accounted for 17.3% of all the adults captured in this study. First detected in Québec in 2001 during WNV mosquito surveillance operations [28], and next detected mainly in the Montérégie region through WNV-oriented surveillance activities, its prevalence and distribution were also certainly underestimated (accounting for only 2.6% of all specimens in 2016 surveillance) [6]. Indeed, *Oc. japonicus* is adapted to colder temperatures: it currently finds suitable habitat conditions in the most temperate regions of central Europe, the eastern USA, and southeast Canada [35,55]. It can be hypothesized that it might be more widely distributed in Québec, over and above the southern regions of Montérégie and Estrie. This hypothesis is supported by other studies [54] and by the episodic findings of adults in CDC-light traps since 2003 in Québec regions north of Montréal, namely Outaouais, Laurentides, Mauricie–Centre-du-Québec, and Saguenay–Lac-Saint-Jean [6]. Its role as a primary disease vector in North America is unclear, but its interactions with other established vector mosquito species could impact local disease dynamics [26]. In the United States, *Oc. japonicus*’ vectorial competency has been demonstrated for WNV, eastern equine encephalitis, and other encephalitis causing viruses [26,29,56,57]. It also has potential to be infected with LACV [58,59]. Evidence confirming the presence and abundance of *Ae. japonicus* in Quebec is significant, stressing the need to update the surveillance program to target a wider range of species in addition to the main WNV vectors. 

It is of note that *Oc. hendersoni* and *Oc. triseriatus* are sympatric species in northeastern America and that interspecific hybridization is possible between these sibling species. In the present survey however, no evidence of an interspecies hybrid was documented because adults of these two species are morphologically indistinguishable (proper differentiation requires genetic analysis). Since our strategy was to taxonomically identify adult specimens, the ones that keyed out to either *Oc. hendersoni*/*Oc. triseriatus* were pooled together in our analysis. This approach may have underestimated the real numbers of *Oc. hendersoni*. This limit inherent to our identification methodology was added to the discussions segment (lines 354–361). It would be interesting to explore this further in terms of public health for future projects

### 3.3. Utility of the AMOT Group as Surrogate Species for Invasive Aedes Spp.

Based on Crans’ classification, two species from the AMOT ecological group, *Oc. triseriatus* and *Oc. Japonicus* [34], were not only detected in our study, but also found to be dominant and spatio-temporally constant between 2016 and 2017 (*Oc. triseriatus* more so than *Oc. japonicus*). The habitat preference exploration for *Oc. triseriatus* and *Oc. japonicus,* based on adult and egg samples, did not highlight any strong associations with certain types of more urbanized environments. These associations seemed, nevertheless, stronger for the abundance of data than in terms of presence and absence. These two species therefore seem relatively ubiquitous and adapted to all environments. These traits of preference make them good candidates for a surveillance system targeted at the detection of any mosquito species. We thus propose the use of local AMOT species as a surrogate for invasive exotic *Aedes* in future studies. In other words, *Oc.* presence is suggested here as a potential indicator to be used in combination with other field variables to help predict where and when introduced IMS could survive and potentially establish in the future. This assumption is supported by research data from Giordano et al., which showed that *Ae. albopictus* was detected, both as adults and at juvenile stages, in the city of Windsor, along with other AMOT mosquitoes [8]. However, since, as in our study, the Giordano study was not designed to assess the relationship between those three species, the results need to be interpreted with caution. Further validation is required to strengthen this concept.

The explanation for the temporal stability and the differing spatial distribution between *Oc. triseriatus* and *Oc. japonicus* could be related to the length of time these species were considered endemic in our study region. Indeed, *Oc. triseriatus* was known to be indigenous to this region of North America much earlier than its counterpart. *Oc. japonicus* was, until recently, classified as an invasive species in Canada, and even more so in Québec. Comparable to *Ae. albopictus*, it is a highly invasive Asiatic species that was accidentally introduced to North America by international trade [35]. This could explain its less stable presence when compared to *Oc. triseriatus*. Thus, *Oc. triseriatus*’ presence could be viewed as being a signal of favorable long-term sites for AMOT species in southern Québec and *Oc. japonicus* as being an indicator of favorable sites for an invasive species introduction before its establishment. More data is needed to properly assess whether or not these species have a stable population, and tests could be done to determine if these two species have differed in their distribution changes through time. 

## 4. Materials and Methods 

### 4.1. Study Region Identification and Settings 

**Figure 5:**
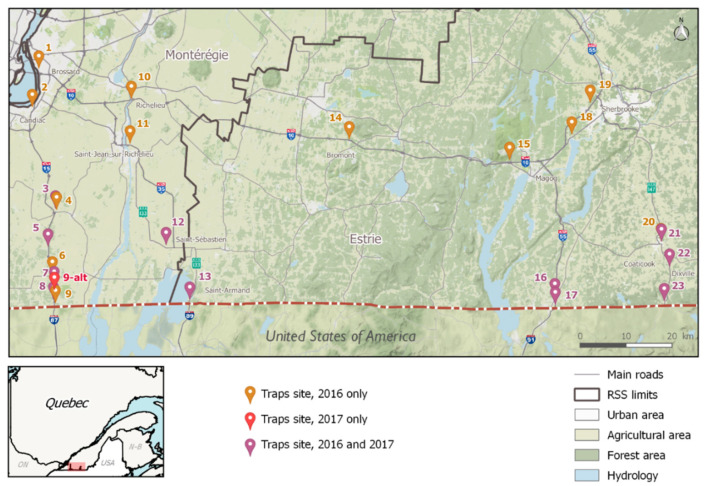
Map of the monitored area and sampling sites in socio-sanitary regions (RSS) in southern Québec, Canada. GPS coordinates are listed in Figure 5.

The study took place during the summers of 2016 (8 August to 29 September) and 2017 (5 June to 28 September) in Montérégie and Estrie, the two southernmost regions of the province of Québec, which share borders with the American states of Vermont and New York (Figure 1). The possibility of vehicles transporting mosquitoes across the Canadian-American border is substantial, considering that considerable ground traffic exists between the two countries. Border controls, gas stations, rest areas, and parking lots (especially truck-adapted ones) located on main connecting roads (entry-points) were targeted as prime IMS introduction sites [42]. Twenty-three sites located along the main four highways used by ground transportation to cross international checkpoints (A-15, A-35, A-55, R-147, as identified by the Québec’s Ministry of transportation) [60] were identified in 2016 as being potential points of entry for the accidental introduction of invasive *Aedes* species (Figure 1). Since the introduction risk of adult mosquitoes posed by ground transport is considered to decrease with distance from the colonized regions, and that drivers are expected to stop or take breaks every other hour on their trips, potential IMS entry-points should be located roughly within a 2.5-h drive from established populations (movement of eggs or immature stages could take them even further) [42]. The southern limit of the study area was on the U.S.-Canada border, approximately 100 km from the northernmost population of *Ae. albopictus* in the United States [10]. The monitoring zone’s limits for the study were: Highway-15 (west), A-55 (east), A-10 (north) and the U.S.-Canada border (south) (Figure 5). Global positioning system (GPS) coordinates were obtained for each site, as well as trapping authorization from landowners. In 2017, only the 12 southernmost sites were visited. GPS coordinates were kept the same for both years, except for site no. 9, which was moved one kilometer north in 2017 for logistical reasons (see Table 2 for GPS coordinates of each sampled sites).

Different, complementary methods were used for mosquito trapping: the fan-operated BG-Sentinel 2 traps (BGS2), designed to be an effective monitoring tool for a wide array of adult mosquito species (including *Ae. albopictus* and other species from the same ecological group) [61], Gravid *Aedes* traps (GAT) supplemented with grass infusion that is also efficient in attracting female *Ae. albopictus*, *Oc. japonicus*, and *Oc. triseriatus* seeking oviposition sites [42], and finally, dark-colored infusion-baited oviposition traps (ovitraps, OVI), which take advantage of the tendency of some mosquito species (including *Ae. aegypti* and *Ae. albopictus*) to use containers as larval development sites, and can be used to detect eggs (indirectly confirming the presence of gravid females) of these species [42]. Each site was divided into four plots, which were located at a distance of 15 m from each other. To capture a maximum number of specimens and enhance the species diversity of each site, three OVI, three GAT, and one BGS2 trap were randomly assigned to each plot for the entire duration of the study (OVI and GAT were paired side by side on three of the plots, with BGS2 standing by itself on the fourth, under the assumption that ecological habitat was homogenous at the site scale). For both years, traps were set for 24 h (± 2 h) at a time, bi-weekly. For simplification purposes, each 24 h capture period was identified as a “capture night” (as traps were set overnight). From 8 August to 6 September 2016, only OVI and GAT were deployed in the 23 sampling locations due to BGS2 manufacturing delays. BGS2 were added from 6 to 29 September 2016. All three trap types were set during the entire 2017 study period (5 June 5 to 28 September). 

### 4.2. Mosquito Sampling and Processing

#### 4.2.1. Eggs

In 2016, 69 OVI were deployed over the 23 sites (three OVI per site) for four different periods of 24 h, with a total trapping effort of 276 OVI-nights. In 2017, 36 OVI were deployed over 12 sites (three OVI per site) for nine periods of 24 h, for a total trapping effort of 324 OVI-nights. OVI are designed to capture mosquito eggs using 4 L spray-painted black plastic buckets with overflow holes that reach maximum capacity at the 2 L mark. Each OVI was filled with 1 L of a natural attractant (2.5 g organic rabbit food pellets, fermented at room temperature in 1 L of tap water for 7 days prior to use) [62]. The lure was used for 24 h and then discarded. Thin, commercial firewood pieces were used as oviposition substrates, standing half-submerged in the attracting solution. All pieces were equal in size, ensuring that each location presented the same surface area for eggs.

Eggs observed on oviposition substrates were brought to the lab and counted using a trinocular magnifier (×80). They were then placed in a hatching solution (1 L) of boiled or carbon-filtered tap water under controlled conditions (100 W lighting, photoperiod of 14 h light: 10 h dark, at 27 °C). The eggs were monitored daily to control hatching conditions (temperature, and relative humidity percentage, etc.), avoid contaminants (e.g., fungal development), and detect hatching. Larval rearing was performed in the same container by adding specific nutrients (yeast, fish food, oats, and liver powder) to the hatching tap water. Specimens were taxonomically identified once they reached the fourth-instar larval stage (2016) or when they emerged as adults (2017, regardless of gender) using an ×80 magnifier and morphological keys [63,64]. 

Unhatched eggs were sent to the National Microbiology Laboratory in Winnipeg, Manitoba for bio-molecular identification, which was performed using sequence analysis of the 5′ end of the mitochondrial gene cytochrome C-oxidase subunit I (CO1) [65]. Nucleic acid (DNA) was extracted from individual unhatched eggs using the QIAamp^®^ DNA Mini Kit (Qiagen (Canada) Inc., Toronto, Ontario, Canada), and a 658 base pair fragment of the CO1 gene was amplified using primers LCO1490 and HCO2198. Reaction mixtures were prepared using the Invitrogen Taq DNA polymerase kit, so that each 50 μL reaction contained: 5 μL of 10× reaction buffer, 0.2 mM of each dNTP, 3 mM MgCl_2_, 0.3 μM of each primer, 2.5 U recombinant Taq polymerase, and approximately 10 ng of DNA template. Amplification of the CO1 gene was carried out with the following temperature cycling parameters: denaturation at 94 °C for 2 min, 30 cycles of amplification at 94 °C for 30 s, 53 °C for 30 s, and 72 °C for 80 s, followed by a 5 min extension at 72 °C. Conventional PCR assays targeting the internal-transcribed-spacer 1 (ITS1) and 2 (ITS2) [66,67], as well as a real-time PCR assay based on species-specific odorant receptor genes [68], were performed as previously described and used to differentiate *Ae. albopictus* from *Ae. aegypti*. Amplification products were analyzed with ethidium bromide-stained 2% agarose gels. The amplification products from conventional PCR were purified using the Promega Wizard SV GL and PCR clean-up system, sequenced on an ABI 3130xl Genetic Analyzer using BigDye™ Terminator version 3.1 cycle sequencing kits. DNASTAR Lasergene 9 Software was used to edit the sequence data, which was subsequently compared to GenBank data.

#### 4.2.2. Adults

In 2016, BGS2 were deployed on the 23 sites (one BGS2 per site) over two nights in September for a total trapping effort of 46 trap-nights. GATs were deployed on the 23 sites (three per site) over four nights in August and September, for a total trapping effort of 276 trap-nights. In 2017, BGS2 and GAT (one and three per site, respectively) were deployed over nine nights from June to September, for a total trapping effort of 108 and 324 trap-nights, respectively. 

GAT traps were used to capture gravid *Aedes* females following the manufacturer’s instructions [69]. Traps were filled with 3 L of the same fermented lure mentioned earlier for OVI. Canola oil was used on the inside wall of the trap as physical insecticide (stickiness). Specimens were collected on site using soft entomological forceps, transported, stored in Eppendorf tubes at −20 °C, and taxonomically identified as previously described.

BGS2 traps were used to capture adult *Aedes* species mosquitoes following the manufacturer’s instructions [70] using the BG-Lure provided with each trap only (changed annually, without adding CO_2_ or octenol). The specimens were trapped in nets provided by the manufacturer and frozen at −20 °C in the lab. The specimens were subsequently identified using the same approach mentioned earlier. 

### 4.3. Species Diversity Assessment

Calculated OVI indices included the OVI positivity index (OPI) (percentage of OVI with evidence of eggs) and the mean egg index (mean number of eggs for all inspected OVI) [71,72]. The relative frequency of eggs over all sites was calculated by species (*Oc. triseriatus*, *Oc. japonicus,* and other species), by month, and by year. Similar indices were calculated for BGS2 and GAT based on the MosquiTRAP positive index developed by Resende (2013), which included: an adult trap positivity index (ATPI; the percentage of BGS2 and GAT with captured adult mosquitoes assessment based on traps where at least one adult was collected) and a mean adult index (mean number of adults for all inspected BCS2 and GAT). A mosquito species richness index (MSRI) was calculated to document the number of different species captured per year for each of the three different traps during the sampling period [73]. MSRI was used to compare the traps’ relative capacity to capture the widest range of different species as possible. Adult mosquito relative abundance was expressed as a percentage of any given species relative to the total sample. The following abundance (density = D) classes were used [74,75]: satellite species (D < 1%), subdominant species (1 ≤ D ≤ 5%), and dominant species (D > 5%). The average relative abundance over all the sites was calculated by species (*Oc. triseriatus*, *Oc. japonicus*, and other species), by month, and by year. Adult mosquito distribution was determined as the percentage of sampling sites at which a given species was documented. The following adult distribution classes were adopted [75]: C1—sporadic appearance (constancy 0–20%), C2—infrequent (20.1–40%), C3—moderate (40.1–60%), C4—frequent (60.1–80%), and C5—constant (80.1–100%). Temporal variation in the presence of eggs or adults of the species *Oc. japonicus* or *Oc. triseriatus*, respectively, from one year to another at the same site was assess using Fisher’s exact test.

### 4.4. Land Use and Land Cover Data

Data on different environmental variables including land use and land cover variables (namely surface water, forests, wetlands, buildings and artificial structures, roads and railroads) were also obtained for each site from the following databases: the Annual Space-Based Crop Inventory for Canada [76], Canadian Wetland Inventory [77], 2005–2010 20 m Land Cover of Canada South of Treeline [78], National Hydro Network [79], National Railway Network [80], and the National Road Network [81]. Based on vector average flight distance and other ecological factors, one-kilometer-radius buffers were created around the centroid of each site to estimate the values of the environmental determinants [82]. These values were calculated either as the proportion of areas occupied by each variable in the buffer (land use and land cover variables) or a total of linear meters (roads and railroads), depending on the geographic feature assessed. Geographic information system (GIS) was used for data extraction and analysis (ESRI ArcGIS, v. 10.4 or above). Five main variables were built, based on their known impact on mosquito biology (affecting life cycle by influencing access to food, shelter, reproduction, and egg-laying sites, etc.): “vegetal cover” is the percentage of forested, shrubby, and agricultural lands that comprise the sites; “developed zones” represents the non-vegetal cover of the sites expressed as a percentage, including surfaces occupied by buildings, hard surfaces, urban areas, parks, industrial sites, factories, and farms; “wetlands” represent the percentage of waterlogged zones, including the ones with temporary vegetation; “surface water” is the percentage of the sites covered by lakes, basins, rivers, and ponds; and “roads network” is the total length (in meters) of segments of highways, service lanes, and roads.

To estimate the association (risk ratio) between species richness and the environmental variables of each of the 24 sites, we used Poisson regression on the OVI and GAT data collected in August and September of 2016 and 2017. To estimate the association (risk ratio) between the number of captures of the AMOT species *Oc. triseriatus* and *Oc. japonicus* and the environmental variables of each site, we used Poisson regression on the OVI and GAT data collected in August and September of 2016 and 2017. To estimate the association (odds ratio) between the presence or absence of *Oc. triseriatus* and *Oc. japonicus* and the environmental variables of each site, logistic regression was used on the GAT and OVI data collected in August and September of 2016 and 2017. To account for repeated measures of the same site over 2016 and 2017 in both Poisson and logistic regression analyses, a generalized estimating equation (GEE) model, using an exchangeable correlation matrix, was applied. R software (v. 3.3.0, R Core Team, 2013. R: A language and environment for statistical computing. R Foundation for Statistical Computing, Vienna, Austria. Available online at http://www.R-project.org/ (accessed on 7 August 2021).) and SAS software (v. 9.4, SAS System for Windows, copyright 2020, SAS Institute Inc.) were used to perform these analyses.

## 5. Conclusions

Since the Zika virus epidemics in the Americas, the arrival of invasive *Aedes* spp. in North America has raised concerns, including for Québec’s public health authorities. Models based on temperature, precipitation, and winter survival capacity predict that under the current climate, southern Québec could be favorable for the temperate *Ae. albopictus*’ establishment (least conservative scenario) and a much greater range expansion could occur for 2011 to 2040 [83]. This study highlights the importance of choosing the appropriate methodology in line with the initial monitoring objectives. To optimize IMS monitoring for the identification of invasive *Aedes* species introduction in southern Québec, based on what has been learned from AMOT species, all three types of traps used in this study should be used minimally from mid-July to mid-August in lightly-dense forested areas with ground transportation activities. Sites could be chosen according to the presence of *Oc. japonicus*. For optimal identification of captured eggs, the fourth-instar larvae method should be employed. Our finding of the presence of *Ae. aegypti* is not a sign of elevated disease risk for humans, but confirms the possibility of a mechanical introduction of an invasive *Aedes* spp. through ground transportation activities in Québec. The repetitive findings of *Ae. albopictus*’ eggs in the neighboring state of Vermont in 2019 and 2020 [84,85] illustrates that the threat of this species is real, and is progressively making its way northward. It is not a question of “if” or “how,” but rather “when” and “what” regarding the impact it is going to have. Finally, the presence of *Oc. triseriatus* and *Oc. japonicus* in southern Québec is of public health significance, since they are competent vectors of a number of zoonotic pathogens. While their presence was known to health authorities, their abundance and spread, as shown in this assay, was unexpectedly high. This should be considered in the province’s risk assessment plan of arboviruses, namely for LACV. Using AMOT species as a surrogate in future projects could prove to be a novel way of approaching the question about the risk of the emergence of vector-borne diseases resulting from climate changes in Canada, given the absence of *Ae. albopictus* occurrence in the province. By providing indicators of suitable habitat for introduction, such an innovative public health preparedness approach could optimize sampling strategies when conducting mosquito surveillance of IMS under climate change. The present study adds to the increasing number of studies reporting range expansions of IMS, and demonstrates that ongoing monitoring, using diversified capture techniques to target a wide range of species, may provide useful information to public health with respect to the growing risk of emerging mosquito-borne diseases in southern Canada.

## Figures and Tables

**Table 1:** Mosquito species documented in 2016 and 2017 using adult traps and OVI.

Species	2016					2017				
	n ^1^ Adults Identified (GAT ^2^, BGS2 ^3^)	n Adults Identified (OVI ^4^) *	n Eggs Hatched to Larvae Identified (OVI)	n Unhatched Eggs Identified (OVI)	Total	n Adults Identified (GAT, BGS2)	n Adults Identified (OVI) *	n Eggs Hatched to Larvae to Adults Identified (OVI)	n Unhatched Eggs Identified (OVI)	Total
*Anopheles punctipennis*	2	0	0	0	2	0	0	0	0	0
*Anopheles quadrimaculatus*	1	0	0	0	1	0	0	0	0	0
*Ochlerotatus aurifer*	3	0	0	0	3	0	0	0	0	0
*Ochlerotatus cantator*	2	0	8	0	10	0	0	0	0	0
*Ochlerotatus hendersoni*	2	1	0	0	3	0	0	0	0	0
*Aedes canadensis*	0	0	0	0	0	30	0	0	0	30
*Aedes intrudens*	0	0	0	0	0	2	0	0	0	2
*Aedes provocans*	0	0	0	0	0	5	1	0	0	6
*Culex tarsalis*	0	0	0	0	0	1	0	0	0	1
*Culex territans*	0	0	0	0	0	2	0	0	0	2
*Coquilettidia perturbans*	0	0	0	0	0	1	0	0	0	1
*Culiseta morsitans*	0	0	0	0	0	1	0	0	0	1
*Ochlerotatus communis*	0	0	0	0	0	4	0	0	0	4
*Ochlerotatus euedes*	0	0	0	0	0	1	0	0	0	1
*Ochlerotatus stimulans*	0	0	0	0	0	9	0	0	0	9
*Ochlerotatus ecrucians*	0	0	0	0	0	1	0	0	0	1
*Aedes cinereus*	8	0	0	0	8	5	0	0	0	5
*Aedes vexans*	6	0	20	0	26	2	0	0	0	2
*Culex pipiens*	35	1	0	0	36	64	1	1	0	65
*Ochlerotatustrivittatus*	2	0	0	0	2	8	0	0	0	8
*Ochlerotatus japonicus*	24	0	109	0	133	42	0	921	41	1004
*Ochlerotatus triseriatus*	21	2	68	84	175	97	0	142	57	296
*Ochlerotatus punctor*	0	0	0	1	1	0	0	0	0	0
*Aedes aegypti*	0	0	0	0	0	0	0	0	1	1
TOTAL:	106	4	205	85	400	275	2	1063	99	1439

^1^ n: quantity, ^2^ GAT: Gravid *Aedes* traps, ^3^ BGS2: Biogent Sentinel 2 traps, ^4^ OVI: * Fortuitous captures of adults in OVI.

**Table 2:** GPS Coordinates of each sampling locations.

SITE ID	GPS Location	SITE ID	GPS Location
**SITE 1**	45.47067, −73.49517	**SITE 12**	45.12438, −73.14796
**SITE 2**	45.3955, −73.51247	**SITE 13**	45.01603, −73.08362
**SITE 3**	45.19625, −73.44946	**SITE 14**	45.33145, −72.64882
**SITE 4**	45.19424, −73.44623	**SITE 15**	45.29167, −72.21335
**SITE 5**	45.12104, −73.46984	**SITE 16**	45.02442, −72.08749
**SITE 6**	45.06675, −73.45808	**SITE 17**	45.00495, −72.08673
**SITE 7**	45.04808, −73.45299	**SITE 18**	45.34148, −72.04212
**SITE 8**	45.01715, −73.45526	**SITE 19**	45.4033, −71.99241
**SITE 9**	45.00901, −73.45091	**SITE 20**	45.13209, −71.7959
**SITE 9-alt**	45.03602, −73.45238	**SITE 21**	45.13073, −71.79746
**SITE 10**	45.41125, −73.24272	**SITE 22**	45.08189, −71.77567
**SITE 11**	45.32447, −73.24624	**SITE 23**	45.01337, −71.7906

**Table 3:** Risk ratios from the Poisson regression analysis of species richness of the sites (OVI eggs and GAT adult data, August and September 2016 and 2017) across large scale environmental variables (land use and land cover variables).

Land Use and Land Cover Variable	Risk Ratio	CV	*p*-Value
Vegetation	1.05	8.0%	0.57
Developed zone	0.66	9.3%	<0.001
Wetlands	1.12	4.3%	0.009
Water	0.87	26.0%	0.60
Roads	0.44	20.9%	<0.001

**Table 4:** Risk ratios from the Poisson regression analysis of the number of adult AMOT species captures for each site (GAT adult data, August and September 2016 and 2017) across large scale environmental variables (land use and land cover variables). The (**a**) association between environmental variables and the number of adults *Oc. triseriatus* captured between August and September 2016 and 2017 with GAT traps; and the (**b**) correlation between environmental variables and the number of *Oc. japonicus* adults captured between August and September 2016 and 2017 with GAT traps.

(a)			
Land Use and Land Cover Variable	Risk Ratio		*p*-Value
Vegetation	0.81	(0.59–1.11)	0.19
Developed zone	0.46	(0.28–0.75)	0.002
Wetlands	0.87	(0.50–1.51)	0.61
Water	0.89	(0.30–2.67)	0.83
Protected areas	0.00	N/A	0.00
Roads	0.60	(0.31–1.15)	0.12
(**b**)			
**Land Use and Land Cover Variable**	**Risk Ratio**		***p*-Value**
Vegetation	1.32	(0.98–1.77)	0.07
Developed zone	0.82	(0.67–1.00)	0.05
Wetlands	0.96	(0.69–1.34)	0.81
Water	0.89	(0.30–2.67)	0.83
Protected areas	1.20	(0.64–2.25)	0.58
Roads	0.39	(0.18–0.84)	0.02

**Table 5:** (**a**,**b**) Odds ratio from logistic regression analysis of the presence-absence of AMOT species (GAT adults and OVI eggs, August and September 2016 and 2017) across large-scale environmental variables (land use and land cover variables). (**a**) *Oc. triseriatus* (**b**) *Oc. japonicus*.

(a)			
Land Use and Land Cover Variable	Odds Ratio		*p*-Value
Vegetation	0.995	(0.959–1.032)	0.77
Wetlands	0.991	(0.947–1.037)	0.70
Developed zone	0.932	(0.898–0.967)	<0.001
Water	0.980	(0.879–1.091)	0.71
Roads	0.932	(0.873–0.994)	0.03
**(b)**			
**Land Use and Land Cover Variable**	**Odds Ratio**		***p*-Value**
Vegetation	1.011	(0.989–1.033)	0.35
Wetlands	1.013	(1.002–1.023)	0.02
Developed zone	0.973	(0.955–0.999)	0.007
Water	0.992	(0.934–1.054)	0.79
Roads	0.923	(0.874–0.975)	0.004

## Data Availability

Data is available upon request from the authors; the data that support the findings of this study are available from the corresponding author upon reasonable request.

## Abbreviations

AMOT	*Aedine multivoltine group*, *Oc. triseriatus* type
ATPI	Adult trap positivity index
BGS2	Biogent Sentinel-2 trap
CDC	Centers for disease control and prevention
GAT	Gravid *Aedes* trap
IMS	Invasive mosquito species
LACV	La Crosse encephalitis virus
MSRI	Mosquito species richness index
OPI	OVI positivity index
OVI	Ovitrap
WNV	West Nile virus
